# Progress in Fevers

**Published:** 1903-06-27

**Authors:** 


### PROGRESS IN FEVERS.

(*Concluded from page 210.*)

**Small-pox and Vaccination.**—In his recent report to the Surrey County Council, Dr. Seaton,[9] while acknowledging that improved sanitary organisation and administration account in part for the decrease in small-pox in modern times, says, "It must be granted that vaccination is essential to us in preventing the spread of small-pox, and that, in fact, without it we should be in a comparatively helpless position." Whether revaccination, as employed in Germany, could be instituted here is, he thinks, "a question for administrators," but he draws attention to the enormous trouble and expense connected with the supervision of unvaccinated "contacts." The question of revaccination is far more boldly pressed by J. C. McVail,[10] who would place it in the forefront of vaccination legislation, pointing out that logically obligatory vaccination should apply to revaccination as well as to primary vaccination. At present only an epidemic of small-pox leads to revaccination, although the purpose of this measure should be prevention of the occurrence of small-pox. In the German Empire, where revaccination is compulsory among a population of 52,000,000 small-pox has almost reached a vanishing point. Allowing the conscientious objector room, legislation on the lines of revaccination would greatly tend to eliminate small-pox. The age for revaccination should, McVail thinks, except in cases of epidemic, be as late as possible in elementary school life. If employers of labour were to refuse workmen who had not been vaccinated or revaccinated on the ground of their being a potential source of infection to others, opposition would tend to diminish. Rates also would be relieved of the burden of maintaining, for possible needs, small-pox isolation hospitals. Again, the question of the supply of properly-prepared and efficient lymph would be simplified if the demand were more steady and regular and not subject to panic occasions. Some startling testimony to the value of vaccination is supplied by Porto Rico.[11] Small-pox was endemic there during the Spanish occupation. In February, 1899, soon after the Spaniards evacuated the island, there were 3,000 recent cases, and the disease was spreading very fast. Vaccination of practically the whole of the susceptible population, that is, of 860,000 out of a population of 960,000, was accomplished in four months, and by July 1st the disease had practically disappeared. Since then the mortality has been 2 per annum instead of the former yearly average of 621. In the words of the writer (Major Ames), "Can any honest, intelligent person doubt, in the face of these indisputable, easily verified facts, what it was that in four short months drove small-pox from its wide and long-time reign in the island and has since kept it out? Vaccination alone did it." In Porto Rico glycerinated lymph was found to rapidly lose its efficacy on importation into the tropical climate prevailing there. The use of ivory "points" was found more certain and quite safe. In the United States,[12] as in our own country, the fact that deadly epidemics no longer occur leads to much apathy on the subject of vaccination. A committee appointed to consider the subject by last year's annual conference of North American Boards of Health[13] advised, among other things, State production, or at least State-supervised production of vaccine lymph, systematic house-to-house vaccination by certified public vaccinators, and adequate training of medical students in the theory and practice of vaccination. R. S. Thomson and J. Brownlie[14] publish a preliminary note on the parasites of small-pox and chicken-pox. They have observed in small pox and in varicella in smears taken from vesicles and pustules and, in hæmorrhagic small-pox, in the blood and hæmorrhagic portions of the skin and the adjacent lymph spaces, small spherical highly-refracting bodies simulating small fat globules. They do not stain with osmic acid, however, and most of them are very refractory to the usual basic or acid stains. They are larger than hæmoconia or streptococci, and appear to be distinct from the ordinary histological or pathological constituents of the blood. They appear to be intercellular. In papers recently communicated to the New York Pathological Society [15] it is stated that "vaccine bodies" are frequently altered red blood corpuscles engulfed by epithelial cells, but this explanation does not agree with all the facts. Vaccine, crushed, concentrated, and filtered, was injected into calves and rabbits. It failed to take. Pyogenic organisms in proportion to the severity of the case were found by another investigator in the heart's blood of 29 cases of fatal small-pox in all cases. J. T. Neech,[16] encouraged by the success obtained in aborting boils by the local application of pure carbolic acid, has applied the same treatment to two cases of small-pox with apparently gratifying results. The vesicles dried up without becoming pustular, secondary fever was averted, constitutional symptoms greatly improved, and pitting was avoided.

**Scarlet Fever.**—The subject of the use of anti-streptococcus serum in scarlet fever [17] is one to which attention has recently been given. Since the discovery of streptococcus by Ogston, the unity of the organism has been affirmed or denied by various observers, such as Behring, Marmorek, and Meyer. Class, of Chicago, first called attention to the relationship of cocci and scarlet fever and demonstrated a large diplococcus often present in the throats of scarlet-fever patients. The scarlet-fever serum recently prepared by Moser, of Vienna,[18] is stated to be the product of cultures of this organism. Satisfied that Marmorek's anti-streptococcus serum was useless in scarlet fever, although three successful cases are reported by Perez,[19] Baginsky, in conjunction with Aronson, of Berlin, directed his attention to the subject. He found streptococci absent from the pharynx in five cases only out of 701 scarlet fever patients examined, while in a large number of *post-mortem* examinations streptococci were demonstrated in the heart's blood, in all the organs, and in the bone marrow. Aronson found practically no morphological difference between the various streptococci obtained from scarlet fever angina, erysipelas, diphtheria, and acute rheumatism, etc. By passage through animals he obtained such a highly virulent culture of streptococcus that the intra abdominal injection of one hundredth million of a c.cm. killed white mice in 36–48 hours. Death did not occur with intravenous injection, showing that the blood could destroy the small number of organisms injected. A filtered culture showed very little virulence, proving that the toxin formation was small and that the poison resided in the bodies of the streptococci. It was found that injection of the filtrate did not protect, while injection of the organisms did protect to a certain extent, especially when given in increasing dose. In horses local reaction, sometimes with the formation of pus, and constitutional disturbance followed these injections. After the injection of scarlet fever streptococci, joint troubles, recalling human acute rheumatism and sometimes ulcerative endocarditis, resulted. Eventually a serum was obtained of which a minute dose was sufficient to protect animals against ten times the lethal dose of a virulent culture of the same organism. Using much larger doses, Aronson was able to cure animals already infected. The action of this serum differs from that of anti-typhoid or anti-diphtheritic serum in that it appears to have no inhibiting effect on the organisms themselves. It causes a slow macroscopic agglutination [20] of the streptococci. The serum of one streptococcus strain, *e.g.* that of scarlet fever, reacts with another, *e.g.* that of acute rheumatism or erysipelas, precisely as it does towards its own strain. This does not necessarily prove, however, that the organism in all cases is identical. Using this serum in a comparatively small number of cases of scarlet fever, Baginsky found only a slight decrease of death-rate, but a marked amelioration of the symptoms. Moser, using his serum prepared in a somewhat similar way, also noticed general improvement and a decided decrease of death-rate. J. Sutherland,[21] writing on the diffusability of scarlet fever, seeks to prove that healthy persons do not ever convey the infection to others, but possibly some of his conclusions are hardly warranted by his premises.[22] Cases of relapse of scarlet fever are recorded by H. Fraser [23] and A. B. Sloan.[24] A. E. Dent [25] reports concurrent scarlet fever and measles in a family of children, each disease being somewhat modified, and J. Cullen [26] describes an interesting fatal case of Hennoch's fulminating purpura during convalescence from scarlet fever. The hæmorrhages were chiefly in the legs.

**Yellow Fever.**—J. W. Ross,[27] in reviewing the brilliant work of the United States Army Commission, refers to his own experiments, conducted on similar lines in the hospital of Las Animas, during winter, in the City of Havana, undertaken to induce belief in the mosquito theory of the transmission of yellow fever in the minds of the medical profession of that city. For close on 140 years prior to the Spanish-American war, Havana had never been free from yellow fever, and the annual number of deaths from the disease ranged from 500 to 1,000, or even occasionally reached 1,600. The imported Spanish soldiers suffered heavily. After the American occupation in 1898, the new sanitary authority were energetic in improving the general sanitary condition of the place. A rapid decrease in the amount of small-pox and other infectious diseases, and an enormous reduction of general mortality followed, but yellow fever was as rife as ever. In February, 1901, Major Gorgas started work on the new theory, devoting himself almost exclusively to the destruction of mosquitos and their haunts, and to the protection from their bites of those stricken with the complaint. From September 27, 1901, to January, 1903, not a single case occurred in Havana, although patients from other parts, protected by mosquito netting, were being constantly carried through the town. No war was waged on fleas, bugs, and other vermin with which the place abounds. The system of quarantine adopted by Major Gorgas is described by himself.[28] The new quarantine regulation requiring protection of the patient from mosquito bites, the exclusion of all intercourse with others not known to be immune, and the subsequent destruction of mosquitoes in the infected house by pyrethrum fumigation, proved more acceptable to the populace than the older, more rigid isolation and the extensive disinfection of fomites then enforced. Stress is laid on the advisability of always endeavouring to make quarantine regulations press as lightly as possible on the people, in order to ensure the co-operation of the populace and the avoidance of concealment. An easy but thorough system of observation of non-immunes is in force among those travelling to the city from neighbouring smaller infected places. The parasite of yellow fever still eludes observation. Agramonte[29] was commissioned to investigate the work of Parker and others at Vera Cruz. The latter discovered in the blood of yellow fever patients a small number of transparent hyaline bodies about half the size of a red blood corpuscle, not motile, but possessing a number of granules showing active Brownian movements. Agramonte also found this body, and at first believed it had some causal relation to yellow fever, but subsequently modified this view on finding the same "parasite" in the blood of people not suffering from the disease.

[9] Ibid., Dec. 6, p. 1785. [10] Ibid., Jan. 10. [11] Med. Rec., Dec. 6. [12] Ibid., Jan. 17, p. 101. [13] Ibid., Nov. 15, p. 788. [14] Brit. Med. Jour., Jan. 31. [15] Ibid., Epitome, Jan. 10. [16] Lancet, Feb. 21, p. 518. [17] Birm. Med. Rev., Jan. [18] Brit. Med. Jour., Nov. 29, p. 1729. [19] Ibid., Epitome, Jan. 10. [20] Med. Rec., Feb. 7. [21] Lancet. Jan. 10. [22] Ibid., p. 114. [23] Ibid., Nov. 29, p. 1458. [24] Ibid., Feb. 14. [25] Brit. Med. Jour., Nov. 15. [26] Ibid., Jan. 24, p. 197. [27] Med. Rec., Jan. 24. [28] Ibid., Jan. 17. [29] Ibid.. Dec. 13.

